# Supplementary material for: Tumour-specific PI3K inhibition via nanoparticle-targeted delivery in head and neck squamous cell carcinoma
Source: Nat Commun. 2017 Feb 13;8:14292. doi: 10.1038/ncomms14292 (PMC5316830; doi:10.1038/ncomms14292)
Supplement: Supplementary Information — Supplementary Figures and Supplementary Tables [file ncomms14292-s1.pdf]

Supplementary Table 1. Characterization of HNSCC PDX models established at MSKCC

PDX	Source	p16	HPV	*PIK3CA*	*TP53*	pAKT	P-selectin
H16	Tonsil	+	-	E542K	WT	+	-
H22	Larynx	-	-	H1047R	R248Q	+	+
H30	Tonsil	+	+	E542K	WT	-	+
H31	UKP	+	+	WT	WT	+	+
H33	BOT	+	-	V344G	WT	+	+

BOT=Base of Tongue

UKP=Unknown Primary

WT=wild type

Supplementary Table 2. Drug content and loading efficiency estimated with F-NMR and UV-Vis

Nanoparticle	Drug content	Loading efficiency
FiBYL719	22%	82%±5%
DexBYL719	20%	79%±4%

Supplementary Table 3.

Complete blood count of mice 24 h following i.v. injection of 25 mg/kg FiBYL719

Parameter	Value	Normal range
WBC	4.34±0.5	1.8-10.7
Hb	13.7±1	11.0-15.1
PLT	824±195	592-2972

Supplementary Table 4. Antibodies used in western blot.

Antibody	Supplier	Catalog #
Phospho-p44/42 MAPK (Erk1/2) (T202/Y204)	Cell signaling	4370S
p-S6 Ribosomal protein (S235/236)	Cell signaling	2211S
p-S6 Ribosomal protein (S240/244)	Cell signaling	2215S
Cleaved PARP (D214) mouse specific	Cell signaling	9544S
Pir (p-IGF-IRbeta (Y1135/1136) InsR beta 19H7	Cell signaling	3024S
Filamin	Millipore	CBL228
phospho-Histone H2A.X (S139), clone JBW301	Millipore	05-636
β-actin (C4)	Santa Cruz	SC47778
p44/42 MAPK (Erk1/2)	Cell signaling	9102
S6 Ribosomal Protein (5G10)	Cell signaling	2217
Phospho-GSK-3β (Ser9)	Cell signaling	5558
Rabbit IgG HRP-linked (secondary)	Cell signaling	7074P2
Bovine anti-mouse IgG-HRP linked (secondary)	Santa Cruz	BO415

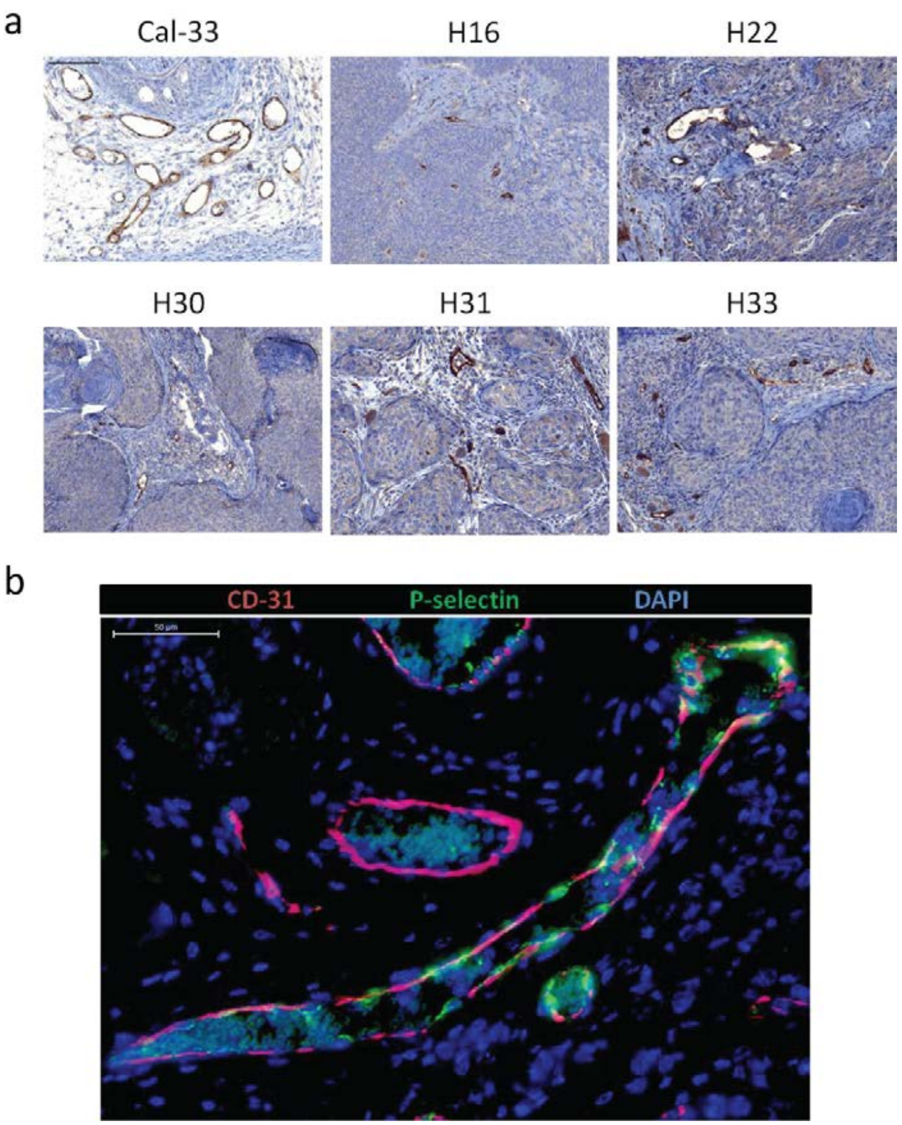

Supplementary Figure 1. P-selectin abundance and p-AKT status in pre-clinical HNSCC models. (**a**) Representative images of immunohistochemistry staining for P-selectin and pAKT in different HNSCC cell lines and patient-derived xenografts (PDX), scale bar, 100 µm. (**b**) Representative images of immunofluorescent staining for CD-31 (red) and P-selectin (green) in H22 PDX. Scale bar, 50 µm.

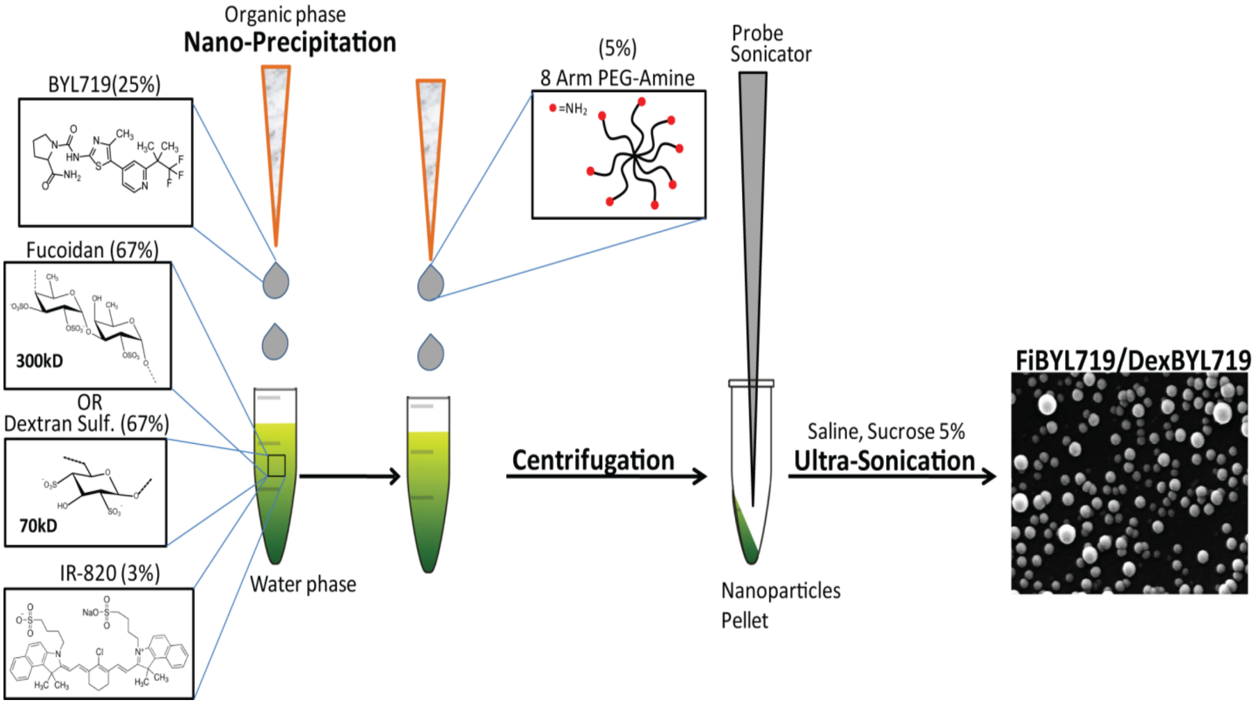

Supplementary Figure 2. Preparation of FiBYL719 nanoparticles using nano precipitation methods.

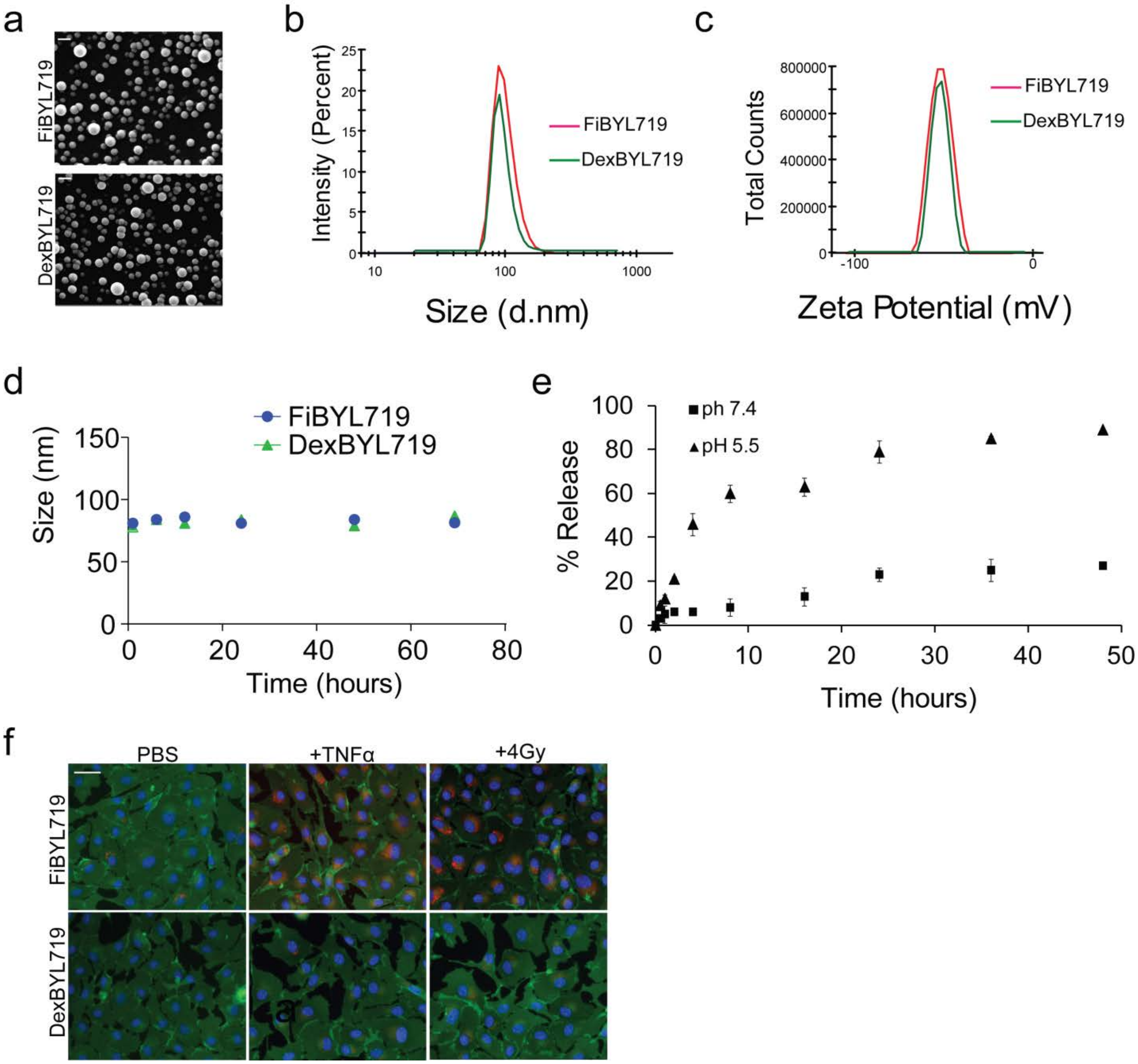

Supplementary Figure 3. Characterization of BYL719-encapsulated nanoparticles. (**a**) Scanning electron microscopy (SEM) images of P-selectin-targeted FiBYL719 and control DexBYL719 nanoparticles. Scale bar = 100 nm. **(b)** Nanoparticle diameters measured with dynamic light scattering (DLS). **(c)** Nanoparticle zeta potential measured with electrophoretic light scattering. (**d**) Nanoparticles stability over time in growth medium containing 10% fetal bovine serum evaluated by dynamic light scattering (DLS). (**e**) Drug release profile of BYL719 from FiBYL719 nanoparticles over time at PBS buffers of pH 5.5 and 7.4. (**f**) Fluorescence images of bovine aortic endothelial cells (BAEC) monolayer treated with either TNFα or ionizing radiation (4 Gy) for induction of P-selectin. Near-infrared dye (red) in FiBYL719 and DexBYL719 nanoparticles. CellMask membrane stain (green). DAPI nuclear stain (blue). Scale bar, 15 μm.

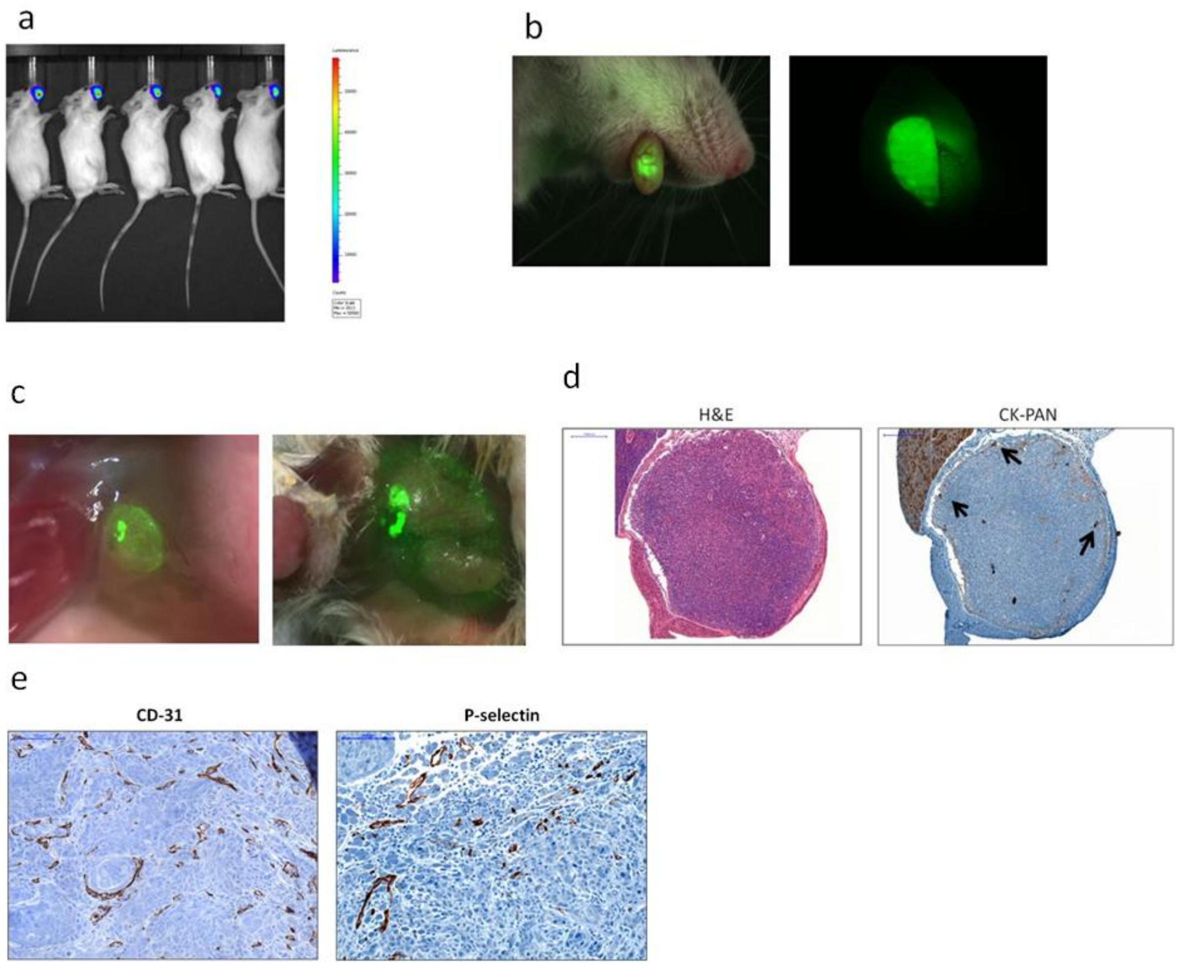

Supplementary Figure 4. Establishment and characterization of orthotopic HNSCC model. (**a**) Bioluminescence imaging of mice 7 days following orthotopic tongue engraftment of GFP-Luc-Cal-33 cells using intraperitoneal injection of D-Luciferin (50 mg/ml). (**b**) *In vivo* imaging of primary orthotopic tongue GFP-Luc-Cal-33 xenograft using fluorescent stereoscopy. **(c)** *In vivo* imaging of cervical lymph node metastasis from orthotopic tongue GFP-Luc-Cal-33 xenograft using fluorescent stereoscopy. **(d)** H&E and immunohistochemistry staining for Pan-cytokeratin (CK-PAN) in cervical lymph node containing metastatic Cal-33 cells (arrows), scale bar, 1000 µm. (**e**) Representative images of immunohistochemistry staining for CD-31 and P-selectin in orthotopic Cal-33 tongue xenograft, scale bar, 100 µm.

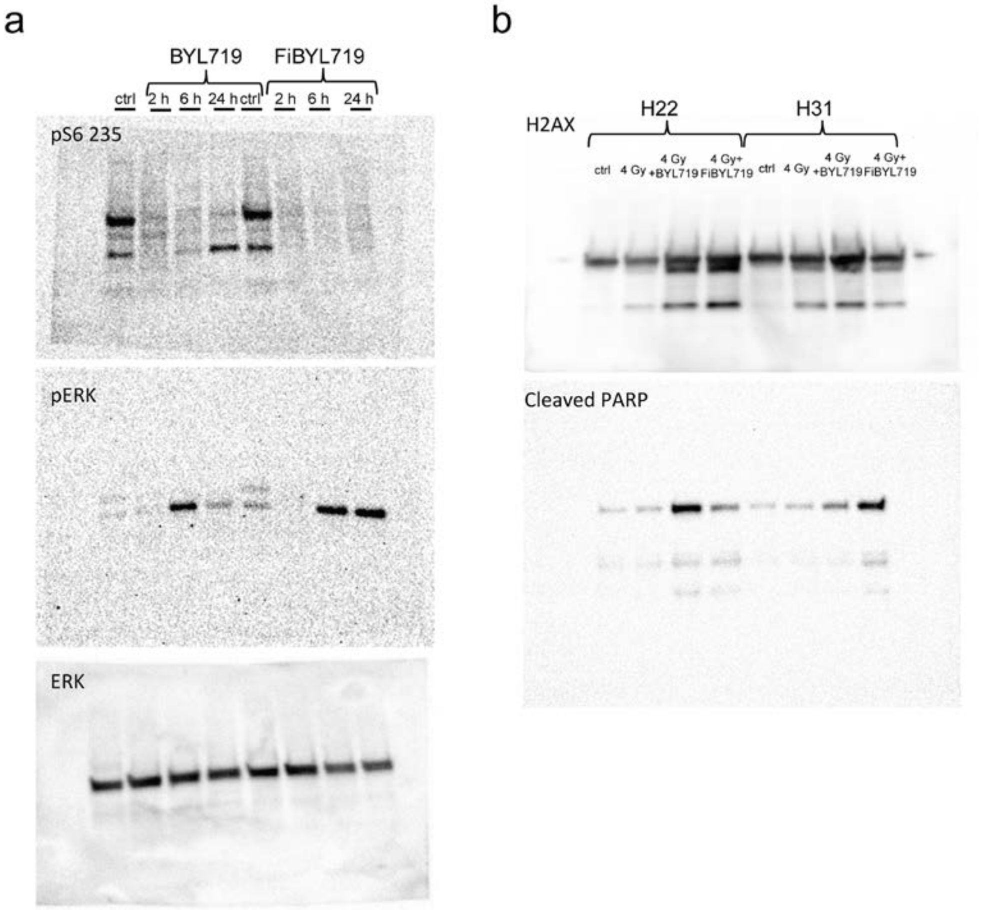

Supplementary Figure 5. Un-cropped western blots from (a) Figure 2, Cal-33 xenografts at different time points following treatment with 25 mg/kg BYL719 or 25 mg/kg FiBYL719, *n*=3. (b) Figure 3, western blot of γH2AX and cleaved PARP in H22 and H31 patient-derived xenografts 24h post treatment with RT (4 Gy) or RT and 50mg/kg BYL719 or 25 mg/kg FiBYL719 (*n*=3).
